# Leaf Sample Size for Pesticide Application Technology Trials in Coffee Crops

**DOI:** 10.3390/plants12051093

**Published:** 2023-03-01

**Authors:** Roxanna Patricia Palma, João Paulo Arantes Rodrigues da Cunha, Denise Garcia de Santana

**Affiliations:** 1 Institute of Animal and Biological Sciences, Universidad Técnica Estatal de Quevedo, Quevedo 120301, Ecuador; 2 Institute of Agrarian Sciences, Federal University of Uberlândia, Uberlândia 38408-100, Brazil

**Keywords:** *Coffea arabica*, ground-based application, airblast sprayers, spray deposition

## Abstract

Plot size, sample sufficiency, and number of repetitions are factors that affect the experimental errors or residuals and the expression of true differences among treatments. The objective of this study was to determine, using statistical models, the appropriate sample size for application technology experiments in coffee crops through the evaluation of foliar spray deposition and soil runoff in the ground-based application of pesticides. In the first stage, we determined the quantity of leaves per set and the volume of the solution for washing the leaves and extracting the tracer. We analyzed the variability between the coefficients of variation (CVs) of the amount of tracer extracted in two droplet classes (fine and coarse), for the different parts of the plants, and for the different quantities of leaves per set that were organized into intervals of five leaves (1–5, 6–10, 11–15, and 16–20). Less variability was found in the intervals with 10 leaves per set and using 100 mL of extraction solution. In the second stage, a field experiment was conducted using an entirely randomized design with 20 plots: 10 sprayed with fine droplets and 10 with coarse droplets. In each plot, 10 sets (samples) with 10 leaves each were collected from the upper and lower canopy of the coffee trees. Moreover, 10 Petri dishes were placed per plot and collected after application. Based on the results of the spray deposition (mass of tracer extracted per cm^2^ of leaf), we determined the optimal sample size using the maximum curvature and maximum curvature of the coefficient of variation methods. Higher variabilities were related to the targets that are more difficult to reach. Thus, this study determined an optimal sample size between five and eight sets of leaves for spray deposition, and four to five Petri dishes for soil runoff.

## 1. Introduction

The statistical accuracy of agricultural experiments for different conditions and crops has been a subject of study over the years. Questions regarding plot size, sample sufficiency, and number of repetitions always exist when designing experiments [1]. This is because these factors are closely associated with the common interest of researchers to obtain the expression of the true differences among the treatments of interest [2] and, from a statistical point of view, they are the main cause of experimental errors or residuals [3].

The methodologies applied to achieve maximum precision, generally use the coefficient of variation (CV) as a measure of experimental precision [4]. Uniformity experiments have been used to study the non-linear decreasing relationship found between the variability measured by the CV (expressed as a percentage) and the plot size, and it is possible to construct a curve that reflects this relationship [5].

Both sample size and number of repetitions affect sample sufficiency, which can concentrate the inherent variability of each plot resulting from factors such as soil, plant architecture, meteorological processes, history of the crops grown in the area, and those obtained from the effects of the treatments, among others. In particular, when there is no difference in the number of samples collected for analysis per experimental unit with the increase or decrease in plot size, the methodologies developed for plot size are more appropriate than those for sample size, as long as the latter does not depend on the former.

Regarding pesticide application technology, given the characteristics of ground-based application trials, collecting equal quantities of leaves and Petri dishes to measure foliar deposition and soil runoff, respectively, is a common practice after application, regardless of their physical size (m^2^). The plot size usually depends on the equipment used for the research and its coverage range. Thus, the size of the experimental plot is determined by the number of observations (samples) made in the field and not by its physical size and, in this case, the terms sampling sufficiency and plot size are synonymous.

Most research concerning the sample/plot size is related to experiments aimed at analyzing the agronomic attributes of different crops, such as their vegetative and productive components, using uniformity experiments. However, few studies have focused on ground-based application technologies. These uniformity experiments mainly consist of plots without a treatment effect (which is why they are called “blank experiments”), where the product obtained from the area is collected in a certain number of small units (0.5–1 m^2^) or basic experimental units (BEU). For these experiments, different plot sizes are formed by the aggregation of adjacent plots [6,7]. In these plots, the same crop practices are maintained for the duration of the experiment [8].

The main difference between uniformity experiments for evaluating agronomic attributes and experiments in the field of pesticide application technology is the blank experiment. Agronomic attributes are evaluated with no required treatment to avoid an additional source of variation. However, in application technology, some treatments are applied to achieve the objectives that are being evaluated. With regard to this, it is recommended that the optimal sample size is determined using randomized designs over a larger area [9] and agronomic attributes [10].

Spray deposition can be evaluated for natural and artificial targets, using tracers and techniques such as spectrophotometry, fluorimetry, and chromatography for its detection [11]. When the natural targets are leaves and the measurement technique is spectrophotometry, sampling should consider two factors: the number of leaves per group, which represents the BEU size, and the number of groups that represent the sample size. Depending on the dose of the tracer (g ha^−1^) used for field application and the amount of extraction solution (mL), one leaf might not contain a concentration sufficient for detection by absorbance. Alternatively, many leaves in the same set could contain such a high concentration, making a dilution necessary.

Although several studies have determined the plot sizes, sample sizes, and number of repetitions in coffee (*Coffea arabica* L.) crops [12,13], it is not possible to determine the optimal plot/sample size for all agronomic traits. The optimal sample size will depend on the crop and its inherent characteristics [3], the treatments and variables [9], the size of the basic experimental or sampling unit [14], and the statistical model [9,15]. Thus, it is recommended that more than one statistical model is used to determine the optimal plot or sample size [16].

Moreover, the International Standard ISO 22522 [17] is applicable to the field measurement of quantities of spray deposit, applied using ground sprayers, on tree and bush crops. It covers measurements of the volume or mass of spray deposits on target structures such as leaves, fruits and ground losses. However, there is not a complete detail of the ideal sample size.

Within this context, the objective of this study was to determine, using statistical models, the appropriate sample size for application technology experiments in coffee crops by evaluating foliar spray deposition and soil runoff in the ground-based application of pesticides.

## 2. Results

### 2.1. Basic Experimental Unit Size

Although the analyses were performed separately for each class of droplet, other parameters such as the plant architecture, target position, number of leaves per set, and volume of the extraction solution could have affected the amount of tracer that was extracted from the leaves under the same application conditions. Thus, in the application with fine droplets, more uniform coverage was obtained in both parts of the plant and the amount of tracer that was extracted only depended on the volume of the extraction solution (Table 1).

For the coarse droplet applications, the amount of tracer extracted from the leaves in the upper part was affected by the number of leaves, possibly due to low deposition. In the lower part, even for the coarse droplet application, the amount of tracer depended on the interdependence between the number of leaves per set and the volume of the extraction solution used to wash the leaves.

**Figure 1:**
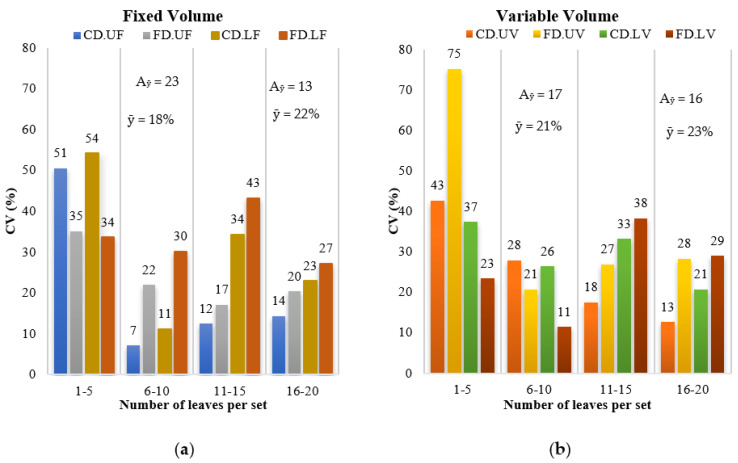
Coefficients of variation (CVs) of the amount of tracer for each number of leaves per set within each droplet size, plant canopy, and fixed (**a**) and variable (**b**) volume of extraction solution in coffee crops. CD: coarse droplets, FD: fine droplets, U: upper canopy, L: lower canopy, F: fixed volume, V: variable volume; A_ȳ_: amplitude; ȳ: average.

The difficulty in washing the leaves with a low volume of the extraction solution or a highly variable concentration when using a fixed volume of solution caused a high CV for the amount of tracer that was extracted in the interval of 1–5 leaves per set for the two droplet sizes and the complete plant profile (Figure 1). The CVs were 51% and 54% in the upper and lower parts, respectively, in applications with coarse droplets and 35% and 34% in applications with fine droplets when a fixed volume was used. With a variable volume, the CVs were 43% and 37% in the upper and lower parts, respectively, for application with coarse droplets and 75% and 23% for application with fine droplets.

For the 6–10 and 16–20 leaf intervals, the variability in the amount of tracer was lower. For the 6–10 interval, the mean CVs between the parts of the coffee plant were 18 and 21% for fixed and variable volumes, respectively (Figure 1). For the 16–20 interval, the averages were 22% and 23% under the same conditions. The smallest amplitudes between the minimum and maximum CVs within the same interval were obtained for the variable volume, with 17% and 16% for the 6–10 and 16–20 leaf intervals, respectively. Thus, as the two intervals had similar CV values, the smaller (6–10 leaves) was selected and the basic unit size of 10 leaves per set and 100 mL of extraction solution was determined.

### 2.2. The Optimal Sample Size for Spray Deposition Trials

The spray deposition on coffee leaves was greater and more uniform in the application with fine droplets, obtaining estimates of 0.72 and 0.86 μg cm^−2^ in the upper and lower parts, respectively (Table 2). In the application with coarse droplets, the spray deposition was approximately 50% lower than that with the fine droplets and was mainly concentrated in the lower part, with a spray deposition of 0.38 and 0.52 μg cm^−2^ in the upper and lower parts, respectively. According to these foliar depositions, the soil runoff obtained higher estimates with coarse droplets (21.16 μg cm^−2^) compared to that with fine droplets (15.06 μg cm^−2^).

The plots did not present with spatial autocorrelation (−0.008 < ρ < 0.578). The optimal sample size for the leaf set (n_0_) for the spray deposition trials on coffee leaves as well as the optimal size for the number of Petri dishes (n_0_) to evaluate the spray soil runoff, varied little between the spatial autocorrelation [18] and exponential [19] models within the same droplet class and plant canopy (Table 2).

The optimal sample sizes for the coffee crops varied between 4.1 and 7.6 leaf sets. In applications with coarse droplets and sampling in the lower part and with fine droplets and sampling in the upper part, the smallest requirements in leaf set were estimated and, for these conditions, low variability was found to be as expected with coefficients of variation (CV_no_) close to 10%.

In the application with fine droplets, the optimal size for determining the soil runoff required three Petri dishes and, under these conditions, the variability estimates were low, with CV_no_ between 6.6% and 6.7%. For the coarse droplets, 4.1–4.2 Petri dishes were required, but the expected variability was a little higher, with CV_no_ between 9.1% and 9.5% (Table 2).

**Figure 2:**
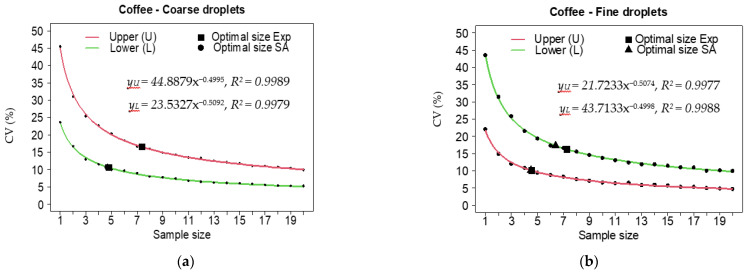
Coefficients of variation (CVs) as a function of the optimal sample size for spray deposition on the leaves for fine (**a**) and coarse (**b**) droplet classes and two parts of the plants, using two statistical models. Sample size in sets of 10 leaves; Parameters a and b significant (*p* < 0.001) in all regressions; Exp: exponential model; SA: spatial autocorrelation model.

**Figure 3:**
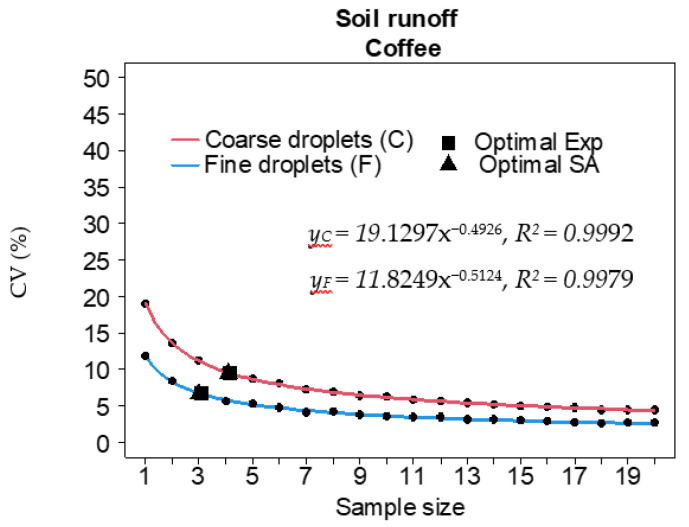
Coefficients of variation (CVs) as a function of the optimal sample size for soil runoff for two droplet classes, using two statistical models. Sample size for the number of Petri dishes; Parameters a and b significant (*p* < 0.001) in all regressions; Exp: exponential model; SA: spatial autocorrelation model.

The two-parameter exponential model was a good fit, with a calculated coefficient of determination above 99% for both droplet classes, for all parts of the plant canopy that were evaluated (Figure 2), and for the soil runoff (Figure 3). In general, the fine droplet class required the largest optimal coffee leaf set sizes (6.4 and 7.3 sets) for samples that were collected from the lower part of the coffee plants and also had the greatest variability in the spray deposition with CV_no_ close to 44%, 55%, and 22%, respectively.

Analysis of the deposition and sample size showed no direct relationship was found between the amount of spray that was deposited on the leaves or soil runoff in relation to the optimal size, likely because there is no way to establish a reference value due to the multiplicity of the involved factors. However, as the amount of spray soil runoff was higher (with averages above 8.55 µg cm^−2^), when not equal, the CVs were smaller than those obtained for the amounts deposited on the leaves. Consequently, the sampling requirement for soil runoff tended to be lower than that for leaf deposition, even though the two quantities are technically distinct.

The sampling recommendation corresponds to the immediate superior integer that was calculated for each model. The CV for the recommended sample size decreased by only tenths of a percent. To facilitate the logistics for subsequent field experiments to evaluate the spray deposition and soil runoff, sample sizes larger than those recommended may be used. The aim should be to collect the same amount of sample in all parts of the plant, but the gain in experimental precision will not be significant for the characteristics with lower sample requirements. However, it is not recommended to use a smaller sample size because it will not have the desired statistical precision.

## 3. Discussion

Crop size, especially its canopy, and droplet class influenced the basic unit size and optimal sample size estimates for the foliar spray deposition and soil runoff trials. For leaf deposition, relatively low values of spray deposition accompanied by high CVs required larger optimal sample size values, while relatively high spray deposition with low variability required smaller sample sizes. As expected, targets more difficult to reach showed higher variability, such as the upper part of the coffee tree, especially when sprayed with the coarse droplet class.

The optimal sample size estimate is dependent on the basic unit size and the variability between them and should, therefore, be the smallest size possible to not overestimate the sample size [14,20]. In the case of pesticide application technology, the basic unit estimate (the number of leaves per set) depends on the efficiency of the spray deposition and the retention capacity of the leaves. Leaves with a smaller area can retain a greater quantity of solution with less variability [21]. In the present study, the 1–5 leaf interval in the coffee crops showed higher CVs. We suspect that these variabilities are partially due to the different leaf sizes, which, being smaller in quantity, influenced the amount of tracer retained. Leaves from the lower canopy were generally larger, on average each set of 10 leaves had 300 cm^2^, while the one from the top had 267 cm^2^.

Spray deposition is influenced by the application technology used, meteorological conditions, plant architecture, and target depth [22]. Application technology experiments are dependent on the equipment, and this is related to the plant architecture target. Thus, in tree crops such as coffee, hydropneumatic sprayers are used with angles between 0° and 80° in relation to the ground, depending on the height of the plant and the equipment. As for plant height, the most difficult target to reach is usually found in the upper part of the canopy and the internal region. This caused high variability, requiring a greater quantity of leaves per basic unit.

Although an inverse relationship has been established between spray deposition and the CV [23], it is possible that the relationship between the application technology used and the target position has more influence on the variability of the deposition, and consequently, on the optimal sample size. Thus, lower deposition levels can have a higher variability and result in larger optimal sample sizes, or similar deposition can occur among the parts of the plant but have a higher variability for the targets that are more difficult to reach. In these targets, the coarse droplets presented a lower performance when evaluating deposition uniformity, increasing the sampling requirement.

The highest variabilities (a CV between 5% and 45%) caused stabilization of the curve with a larger sample size (eight sets of leaves). Other authors have related the effect of high initial variability to the onset of the regression curve (coefficient a) and the heterogeneity of the samples (coefficient b) [24,25].

The low spatial autocorrelation coefficients (−0.008 to 0.578) reflected the independence between plots that was guaranteed based on the experimental design with the randomization of the experiment. Since there was no spatial dependence effect among samples, the two statistical models resulted in a similar sample size.

Owing to the numerous combinations of parameters that can be used at the time of application, a relationship between these parameters and the spray deposition for different crops and in different layers of the canopy cannot be defined, as there would be different results even for the same crop [26,27]. This is mainly due to the heterogeneity of field studies [28].

Although using the maximum number of samples can ensure some statistical certainty, oversampling can result in a loss of resources and depending on the size of the experiment and the type of experimental material, the unfeasibility of the sample size. Additionally, the smaller the effect of the treatments on the dependent variables, the larger the sample size should be [2].

In this context, the objective of the present study was neither to seek a configuration of the parameters involved in the deposition efficiency, nor to evaluate the deposition, but to study the different variabilities as a function of the different droplet classes and in different parts of the plant canopy. Therefore, the optimal sample sizes reported can be used as a reference for other experimental designs in the field of pesticide application technology.

## 4. Materials and Methods

### 4.1. Characterization of the Area and Equipment

The experiment was conducted on the experimental farm belonging to the Federal University of Uberlândia (UFU) (Municipality of Uberlândia, MG, Brazil). The area has an altitude of 912 m and is located at 18°58′52″ S 48°12′24″ W. The area has slightly undulated topography and the soil is classified as dystrophic red latosol of clayey texture. We analyzed the optimal sample size with two droplet classes and a fixed application rate in a 15-year-old coffee crop, cv. Topázio, with 3.5 m row spacing, 0.7 m between the plants, and an approximate tree row volume of 10,389 m^3^ ha^−1^, measured prior to application.

A Montana ARBO 360 airblast sprayer (Kuhn; São José dos Pinhais, Brazil) coupled to a Massey Ferguson 4 × 2 tractor model 265E (Massey Ferguson; Canoas, Brazil) with 47.8 kW (65 hp) power, were used for application. The sprayer had a polyethylene tank with a 300 L capacity, 12 nozzle holders (6 in each side arch), and manual control of the sessions. The MAG 3 hollow cone nozzle (Magnojet; Ibaiti, Brazil) and the TVI 8002 hollow cone nozzle with air induction (Albuz; Evreux, France) were used to generate fine and coarse droplet classes, respectively. The working pressure was 517 kPa, the application rate was 300 L ha^−1^, and the working speed was varied between 5.8 km h^−1^ and 6.9 km h^−1^ according to the flow rate of the nozzles for fine and coarse droplets, respectively.

During the applications, we monitored the meteorological conditions, including the temperature, relative humidity and wind speed (km h^−1^).

### 4.2. Evaluation of the Basic Experimental Unit (BEU) Size

Before studying the sample size, it was necessary to determine the BEU size for the number of leaves per set. For this, we randomly selected two areas with three rows of 60 m each. The application was performed with a tracer and one area was sprayed with fine droplets and the other with coarse droplets, maintaining the parameters detailed in the previous section.

**Figure 4:**
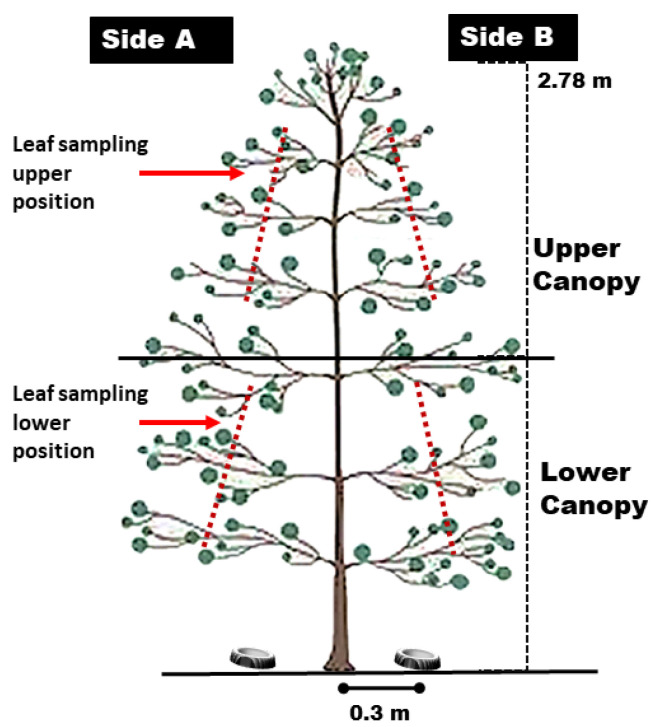
Heights used for sampling the coffee trees (Side A: left, Side B: right).

After tracer application, we collected sets that contained different numbers of leaves. The upper (from the middle of the plant to the top) and lower (from the soil to the middle of the plant) canopy of the plant profile was sampled (Figure 4) and the content of the sets varied between 1 and 20 leaves. Six repetitions were performed for each set, totaling 240 samples (20 sets of leaves × 2 plant positions × 6 repetitions) that were arranged in light isolation for transport to the Agricultural Mechanization Laboratory of the UFU (Uberlândia, MG, Brazil).

For leaf washing, the six replicates were divided into two groups, with three replicates with a fixed volume of extraction solution (distilled water) and the other three replicates with a variable volume. For the fixed volume, 100 mL of solution was used per group, regardless of the number of leaves and for the variable volume, 10 mL was used per leaf. The extraction was performed by grouping all the leaf of each set (20 sets: 1 to 20 leaves).

The Brilliant Blue tracer (internationally cataloged by the Federal Food, Drug, and Cosmetic Act as FD&C Blue No. 1) was added to the spray mixture at a dose of 400 g ha^−1^, to be detected by absorbance in a spectrophotometer (model SP-22; Biospectro; Curitiba, Brazil) regulated at a 630 nm wavelength, which corresponds to the blue range. The extraction of the tracer was performed according to the methodology of Gitirana Neto and Cunha [29] with mechanical agitation of the samples using a pendulum shaking table (model TE240/I; Tecnal; Piracicaba, Brazil), regulated at 200 rpm for two minutes per sample. The leaf areas were determined per set of leaves with a leaf area meter (LI-3100; Li-Cor; Lincoln, USA).

**Figure 5:**
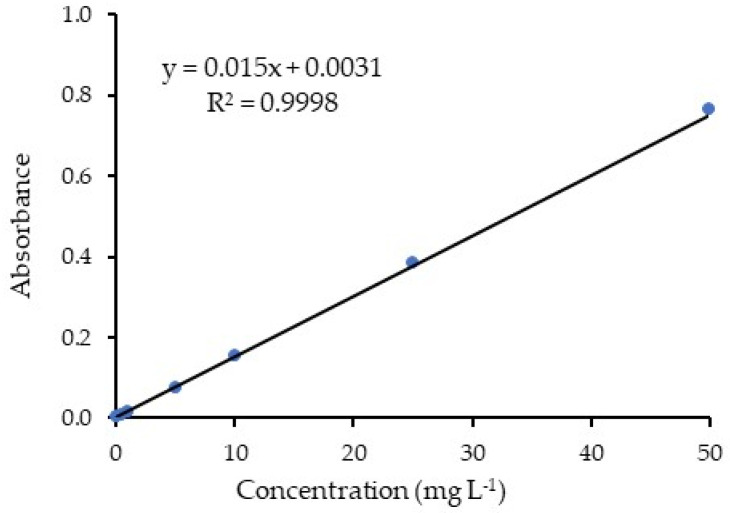
Calibration curve used to determine the tracer amount.

The absorbance values were converted into the tracer concentration in μg L^−1^ using a previously determined calibration curve (Figure 5), performed using solutions of known concentration, and the mass of the deposited tracer was obtained in relation to the quantity of the extraction solution that was used for washing the leaves. Subsequently, the tracer mass was divided by the leaf area of each set of leaves to determine the spray deposition (the quantity of the tracer; μg) per leaf area (μg cm^−2^). Thus, the amount of tracer per cm^2^ of the leaves was obtained for each group for the two volumes (fixed and variable) of extraction solution, for each droplet class (fine and coarse), and for different positions in the plant profile.

To facilitate the statistical analyses, the deposition results for each set of leaves were used to establish intervals according to the number of leaves per set. The sets between 1 and 20 leaves were analyzed in intervals formed by sets of five leaves (1–5, 6–10, 11–15, and 16–20 leaves). An analysis of variance was performed considering the experiments as a randomized 2 × 4 factorial design, with the first factor corresponding to the volume of the extraction solution (fixed and variable) and the second factor being the four intervals (1–5, 6–10, 11–15, and 16–20 leaves), totaling eight treatments with three replicates.

We calculated the CV (%) of the amount of tracer (μg cm^−2^) obtained for the two classes of droplets (fine and coarse) and the different positions in the plant profile (Figure 4) in each interval (1–5, 6–10, 11–15, and 16–20 leaves).

To choose the BEU size (the number of leaves per set), we prioritized the interval of the smallest number of leaves [14] with the lowest average coefficient of variation among the different characteristics of the same interval, together with the smallest amplitude (maximum–minimum) between the CVs for the characteristics of the interval.

For the evaluation of the soil runoff, it was not necessary to modify the basic unit, and we considered a Petri dish as the BEU. We used 30 mL of the extraction solution, and the volume was calculated using previous studies as a reference [28].

### 4.3. Evaluation of the Sample Size

**Figure 6:**
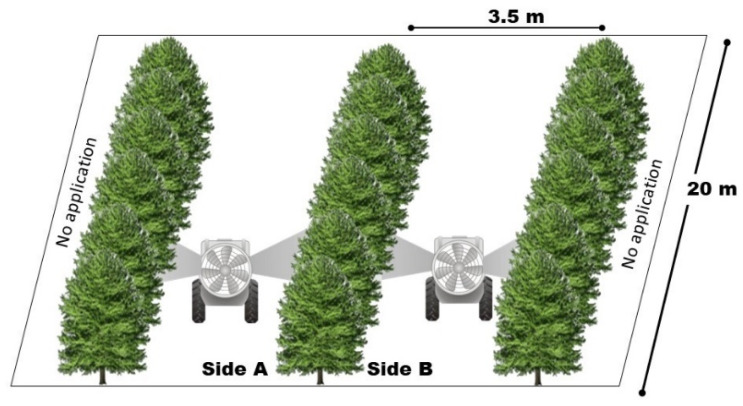
The plot dimensions for the coffee crops.

To study the optimal sample size in coffee crops, we adopted an entirely randomized design with 20 experimental units and 10 plots for each class (fine and coarse). Each plot was composed of three rows of 20 m each, with a total experimental area of 4200 m^2^ and the central row was used for sampling except for the three plants at each end (six plants in total). The analyzed variables were the quantity of extracted tracer in each droplet class (fine and coarse) and in each part of the plant profile (Figure 6).

To determine the optimal sample size for evaluating the spray deposition on the different parts of the coffee tree canopy, after the tracer applications, we collected at the field 10 sets of leaves (sampling units) from each plot (n_i_) and per each part (upper and lower) of the plant profile, with 10 leaves per set. To evaluate the sample size for the soil runoff, we collected 10 Petri dishes per plot which were previously placed under the canopy 30 cm from the coffee tree stem.

The total number of samples was 100 sets of leaves (10 sets per n_i_) per evaluated part and for each droplet class. Regarding soil runoff, the total number of samples was 100 Petri dishes (10 dishes per ni) for each droplet class. After collecting the samples, the leaves and Petri dishes were placed in Styrofoam boxes with light insulation for transportation to the Agricultural Mechanization Laboratory of the UFU (Uberlândia, MG). The extraction of the tracer, both from leaves and Petri dishes, was performed following the procedures that were detailed in Section 4.2. In this case, we used 100 mL of extractor solution per sample.

To eliminate the influence of the variations among individuals for the estimation of the optimal sample size, we calculated the average of the spray deposition on the leaves per unit area (μg cm^−2^) and the soil runoff (μg cm^−2^) of the n_i_. Thus, the total number of samples (100) was reduced to 10 per part of the plant profile and per droplet class.

For the application of the exponential model of Lessman and Atkins [19], in the data analysis process, subsamples were simulated by varying the number of sampling units between 1 and 20 sets of leaves or the Petri dishes with up to a thousand re-samples with replacement. The mean, variance, and CV were calculated among the subsamples of size x. The CVs between subsamples of between 1 and 20 sets of leaves or Petri dishes were regressed using the equation that was proposed by Lessman and Atkins [19]:

(1) y=axb

where *x* is the plot size in BEU, *y* is the CV_(*x*)_ between plots of size *x-BEU*, and *a* and *b* are parameters to be estimated considering for *a* the V_1_ and for *b* the regression of logVxilogxi.

The curve reflects the decreasing relationship between the sample size and the CV for each droplet class and part of the plant profile. From the regression, parameters *a* and *b* were also extracted to calculate the optimal sample size using the equation proposed by Meier and Lessman [30] which corresponds to the maximum inflection point of the curve:

(2) $$X_{c}={\left[\frac{a^{\prime 2}b^{\prime 2}\left(2b+1\right)}{b+2}\right]}^{\frac{1}{2b+2}}$$

where *X_c_* is the point of maximum curvature and the parameters (*a*′) and (*b*′) are calculated using regression.

To apply the spatial autocorrelation model [18], we calculated the spatial autocorrelation coefficient (ρ) among the 10 samples for each part of the coffee tree canopy and each droplet class with the equation:

(3) $$\hat{X_{o}}=\frac{10\sqrt[{3}]{2\left(1-{\hat{\rho }}^{2}\right)S^{2}\bar{Z}}}{\bar{Z}}$$

(4) $$\hat{\rho }=\frac{\sum_{i=1}^{rc}\left(\hat{\varepsilon_{1}}-\bar{\varepsilon }\right)\left(\hat{\varepsilon_{i-1}}-\bar{\varepsilon }\right)}{\sum_{i=1}^{r}\left({\left(\hat{\varepsilon_{i}}-\bar{\varepsilon }\right)}^{2}\right)}$$

where $\bar{Z}$ is the sample mean, $S^{2}$ is the sample variance, $\hat{\rho }$ is the first-order spatial autocorrelation coefficient, estimated by Equation (4), and $\bar{\varepsilon \;}\;e\;\hat{\varepsilon_{i-1}}\;$ are the errors of a model that contains only the intercept in the BEU of *i* and *i*–1, respectively. The CV of the model was calculated with the equation:

(5) $$CV_{\left(x\right)=}\frac{100\sqrt{\frac{\left(1-\hat{\rho }\right)S^{2}}{{\bar{Z}}^{2}}}}{\sqrt{x}}$$

where $\bar{Z}$ is the sample mean, $S^{2}$ is the sample variance, and $\hat{\rho }$ is the first-order spatial autocorrelation coefficient.

Hereby, the appropriate sample size was calculated for the two droplet classes in the two parts of the plant profile using the two statistical models. The analyses were performed using Microsoft Office Excel v. 2203, Sigma Plot 12.0, and R v 4.0.2 software, adopting an α = 0.05 when necessary.

## 5. Conclusions

The basic experimental units for the coffee crops with the least variability was 10 leaves per set and 100 mL of solution to extract the applied tracer.

The spray deposition on the targets was dependent on the plant architecture, collection position, and droplet classes that were used for spraying. Targets with difficult access or with high variability in the deposits required a larger sample size.

The present study defined the optimal sample size for each droplet class and part of the plant canopy. For the coffee crops, between five and eight sets of 10 leaves should be used for evaluating spray deposition, and four to five Petri dishes should be used to evaluate soil runoff.

## Figures and Tables

**Table 1:** A summary of the analysis of variance (F test) for the amount of tracer extracted from coffee leaves as a function of the volume of the extraction solution (fixed or variable) and the number of leaves per sample.

	Coarse Droplets		Fine Droplets	
Source of Variation	Upper	Lower	Upper	Lower
Volume (V)	1.55 ^ns^	1.14 ^ns^	11.77 *	16.89 **
Number of leaves (N)	5.38 *	2.78 ^ns^	2.95 ^ns^	1.68 ^ns^
V × N	1.08 ^ns^	4.06 *	1.55 ^ns^	2.56 ^ns^

* significant (*p* < 0.05); ** significant (*p* < 0.001); ^ns^ not significant.

**Table 2:** Optimal sample size for spray deposition on coffee leaves and soil runoff as a function of droplet size and collection position in the canopy.

Droplet Class	Canopy/Soil	Original Data		Model Data			Recommendation	
		ȳ ± s	CV_ȳ_	Model	^1^n_0_	CV_no_		
		(μg cm^−2^)	(%)			(%)	n_0_	CV (%)
Coarse	Upper	0.38 ± 0.17	45.3	Autocorrelation	7.4	16.6	8.0	16.0
				Exponential	7.4	16.5		15.9
	Lower	0.52 ± 0.12	23.1	Autocorrelation	4.7	10.5	5.0	10.2
				Exponential	4.8	10.6		10.4
	Ground	21.16 ± 4.12	19.5	Autocorrelation	4.1	9.1	5.0	8.3
				Exponential	4.2	9.5		8.7
Fine	Upper	0.72 ± 0.16	21.5	Autocorrelation	4.5	10.0	5.0	9.4
				Exponential	4.6	10		9.6
	Lower	0.86 ± 0.38	44.1	Autocorrelation	6.4	14.2	8.0	12.7
				Exponential	7.3	16.2		15.5
	Ground	15.06 ± 1.75	11.6	Autocorrelation	3.0	6.6	4.0	5.1
				Exponential	3.1	6.7		5.2

^1^ n_0_: optimal sample size for the number of sets of 10 leaves or for the number of Petri dishes, for spray deposition on the leaves or soil, respectively; ȳ: mean; s: standard deviation; CV_no_: coefficient of variation in optimal size; and CV: coefficient of variation.

## Data Availability

All data included in the main text.
